# The Cooperation Between Nurses and a New Digital Colleague “AI-Driven Lifestyle Monitoring” in Long-Term Care for Older Adults: Viewpoint

**DOI:** 10.2196/56474

**Published:** 2024-05-23

**Authors:** Sjors Groeneveld, Gaya Bin Noon, Marjolein E M den Ouden, Harmieke van Os-Medendorp, J E W C van Gemert-Pijnen, Rudolf M Verdaasdonk, Plinio Pelegrini Morita

**Affiliations:** 1 Research Group Technology, Health & Care Saxion University of Applied Sciences Enschede Netherlands; 2 Research Group Smart Health Saxion University of Applied Sciences Enschede Netherlands; 3 TechMed Center Health Technology Implementation University of Twente Enschede Netherlands; 4 School of Public Health Sciences University of Waterloo Waterloo, ON Canada; 5 Research Group Care and Technology Regional Community College of Twente Hengelo Netherlands; 6 Domain Health, Sports, and Welfare Inholland University of Applied Sciences Amsterdam Netherlands; 7 Spaarne Gasthuis Academy Hoofddorp Netherlands; 8 Centre for eHealth and Wellbeing Research Section of Psychology, Health and Technology University of Twente Enschede Netherlands; 9 Research Institute for Aging University of Waterloo Waterloo, ON Canada; 10 Department of Systems Design Engineering University of Waterloo Waterloo, ON Canada; 11 Centre for Digital Therapeutics Techna Institute University Health Network Toronto, ON Canada; 12 Institute of Health Policy, Management, and Evaluation Dalla Lana School of Public Health University of Toronto Toronto, ON Canada

**Keywords:** artificial intelligence, data, algorithm, nurse, nurses, health care professional, health care professionals, health professional, health professionals, health technology, digital health, smart home, smart homes, health monitoring, health promotion, aging in place, assisted living, ambient assisted living, aging, gerontology, geriatric, geriatrics, older adults, independent living, machine learning

## Abstract

Technology has a major impact on the way nurses work. Data-driven technologies, such as artificial intelligence (AI), have particularly strong potential to support nurses in their work. However, their use also introduces ambiguities. An example of such a technology is AI-driven lifestyle monitoring in long-term care for older adults, based on data collected from ambient sensors in an older adult’s home. Designing and implementing this technology in such an intimate setting requires collaboration with nurses experienced in long-term and older adult care. This viewpoint paper emphasizes the need to incorporate nurses and the nursing perspective into every stage of designing, using, and implementing AI-driven lifestyle monitoring in long-term care settings. It is argued that the technology will not replace nurses, but rather act as a new digital colleague, complementing the humane qualities of nurses and seamlessly integrating into nursing workflows. Several advantages of such a collaboration between nurses and technology are highlighted, as are potential risks such as decreased patient empowerment, depersonalization, lack of transparency, and loss of human contact. Finally, practical suggestions are offered to move forward with integrating the digital colleague.

## Introduction

The growing preference of older adults to age in place requires technologies that can help them to do so. One potential technology is artificial intelligence (AI)–driven lifestyle monitoring, based on data collected from ambient sensors in an older adult’s home. However, designing and implementing this technology in such an intimate setting requires collaboration with nurses experienced in long-term and older adult care. This viewpoint paper emphasizes the need to incorporate nurses and the nursing perspective at every stage of designing, using, and implementing AI-driven lifestyle monitoring in long-term care settings. The goal of this collaboration for nurses would be to gain a tool that does not replace them in their role but rather acts as a sort of coworker providing care support and data insights, seamlessly integrating into nursing workflows. Let us welcome this new digital colleague.

While definitions of AI differ, a simple one would be “computers mimicking human behavior” [1]. In the simplest terms, AI is computers learning to interpret large amounts of data and come to conclusions based on that interpretation. AI is essentially a system that gets smarter the more information it is given and uses that knowledge to provide solutions or create products. AI works by taking a large quantity of heterogeneous data, finding patterns in it, and using those learning elements to make more accurate predictions [1]. While AI may appear to be a fairly new technological advancement, its roots actually date back to the 1950s, with its trajectory experiencing various periods of growth and decline over time [2]. In recent years, however, attention toward AI has grown significantly. In particular, so-called generative AI (for example ChatGPT [3], which can “create” outputs such as texts and photos) has received a lot of attention and opened new possibilities [4].

However, the impact of AI is far greater than text and photo generation. AI has been dubbed a general-purpose technology; a game-changer that affects many parts of our lives and industries [5]. Other examples of such breakthrough technologies are the steam engine, electricity, and computers [6]. AI can do many things, from helping doctors diagnose diseases to streamlining business processes. However, as we use AI more and more and increase our reliance on it, we need to make sure we apply it responsibly and intelligently. The growing attention toward AI is also reflected in national and international reports describing AI strategies, with more than 25 countries having developed such a strategy, though views toward AI vary widely around the world [7]. While national AI policies strongly differ from each other, there is generally a lot of attention paid to AI expertise and data policies, whereas attention toward human-computer cooperation has been mostly lacking. In recent years, legislation around AI has received more consideration, with several AI-specific acts and legal frameworks developed around the world [8], focusing on topics such as responsible, validated, and fair data exchange. For instance, the use of AI in the European Union will be regulated by the European Union AI Act [9].

The use of AI leads to a need to consider some broader societal implications including potential downsides. In particular, the prospect of increasing our reliance on automation raises concerns about loss of compassion and humanity in interactions with the subjects of data [10-12]. Indeed, overreliance on algorithms risks increasing bias by a range of societal factors such as age, gender, ethnicity, ability, and socioeconomic status [12-14]. Furthermore, there are privacy concerns: given that AI potentially involves novel uses of sensitive data, there is a need to ensure that this data (and by extension, its subjects) are still protected [15-17].

## AI in Nursing

The added value of AI in health care is evident in various aspects, including the enhancement of health care research equity and versatility, the streamlining of workflows in health care practice, and the personalization of learning within health care education [18]. However, while there is extensive available literature on how AI changes health care in general, the influence on nursing in long-term care is less commonly discussed [19,20]. Especially in more clinical settings, the use of AI is further developed and already being used; for instance, for breast cancer detection in screening mammography [21]. From previous research, the reception of the prospect of a more AI-involved future for nursing has been mixed, with concerns having been expressed regarding the complexity of AI and how its use may affect human interaction and professional autonomy [20,22,23]. This has been especially well-studied among nursing students, who can reasonably expect to see more AI used during their careers [23,24]. Given the relative newness of this area, the precise impact of AI on the nursing field in long-term care is still up in the air. The potential impact includes opportunities for in-home assessment of patients, offering greater time savings and convenience for both patients and health care professionals such as nurses [15,25-28]. This aspect of time savings could also be helpful given the shortage of nurses [29] as the use of technology could potentially reduce the nursing workload [30]. Furthermore, the general provision of more evidence-based, personalized care based on algorithmically derived health information [19,31] can help to overcome intuitively based decisions. The advantage of automation is the ability to take away some of the repetitive drudgery of background work, such as gathering information and administration, as this is handled by the algorithm. Rather, professionals can spend more time on directly action-oriented tasks [31].

## AI-Driven Lifestyle Monitoring System

**Figure 1:**
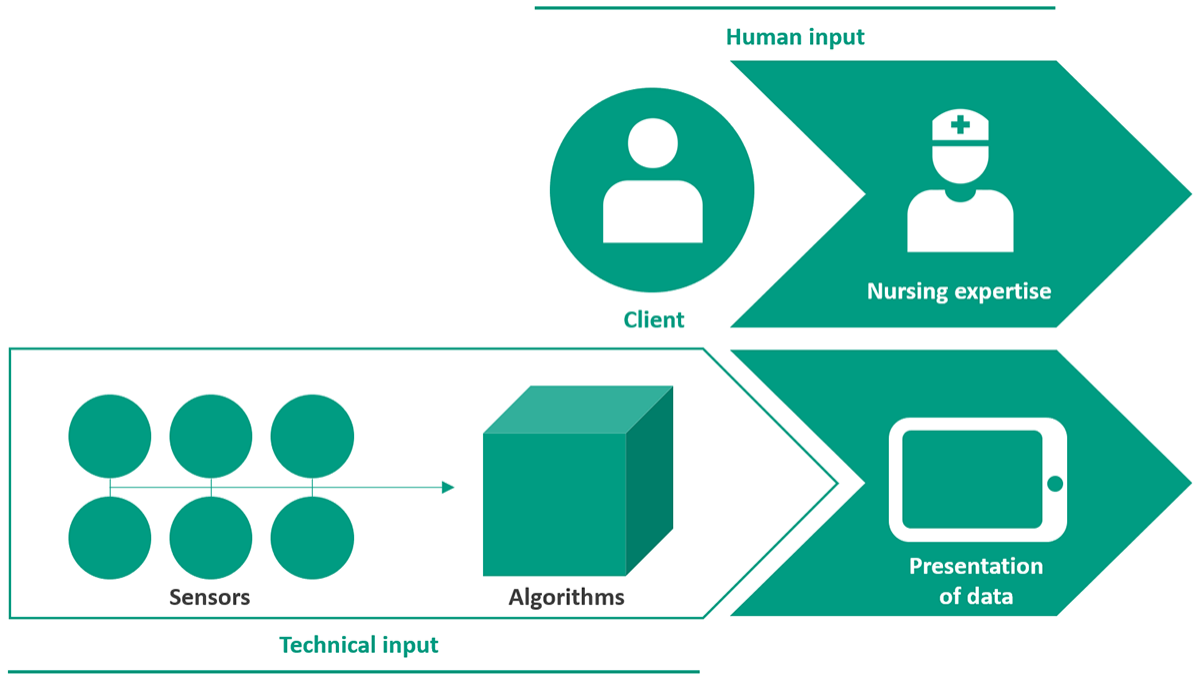
Visualization of the cooperation between nurses and the new digital colleague: artificial intelligence–driven lifestyle monitoring systems.

This paper will focus on the use of AI-driven lifestyle monitoring systems such as those often implemented in smart living environments and smart homes [32]. These systems can be used in long-term care, where over time people might struggle to maintain the basic abilities necessary to keep living well. AI-driven lifestyle monitoring systems are used to obtain insights into a person’s behavior. Examples of such are their daily routine, habits, and activity patterns [25,33,34]. These insights can be used to assist nurses in providing personalized care and support older adults to age in place [15,33,35]. AI-driven lifestyle monitoring systems (Figure 1) work by getting input from ambient and environmental sensors in the home of a person. These can be various types of sensors, such as those for infrared motion, contact, light, temperature, and humidity [36], as well as sensors for physiologic parameters such as heart rate, blood oxygen saturation, and respiratory rate [37]. These sensors monitor the home and the person living there continuously. The combination of the output of those sensors is used by an algorithm to identify patterns and learn what a common lifestyle pattern is for this specific person. While this continuous monitoring takes place, deviations from the common pattern can also be detected [38]. Examples of such deviations include a noticeable decrease in movement, more frequent use of the toilet, or a more restless sleep pattern [36]. The system will then give some form of output (eg, reports or alerts to care providers) by presenting the findings to the user [39]. These findings could potentially support nurses in clinical decision-making, although it is important to include the perspective of nurses when designing these systems, to make the output meaningful for practice [19,40] (Textbox 1).

**The use of artificial intelligence–driven lifestyle monitoring in practice.**

**Case**

Emma Smith, a 53-year-old nurse, is deeply committed to the residents she cares for. She works at a long-term care facility for people with early-stage dementia. Recently, artificial intelligence–driven lifestyle monitoring has been introduced in her department. The residents are monitored using various sensors. Emma finds this both convenient and a bit nerve-wracking. She wonders, “Can I truly rely on the system? What if it misses something?” She also feels slightly uneasy about the sensors taking over a part of her job. However, she appreciates that these tools provide her with a better understanding of the residents’ situations. Every morning, she opens the overview that is generated by the system and sees in one glance if the system has identified any deviations in the metrics of the residents she cares for. Additionally, if there is an incident like a fall, the system immediately sends an alert. “It took me some time to incorporate this in my daily workflow, but now it is part of my routine.”

This technology is of interest due to its potential impact on several trends in nursing that are expected to receive increased attention in coming years: personalized care, aging in place, and positive health. Regarding the first, greater use of AI-driven lifestyle monitoring facilitates understanding of patient health, which in turn can be used to optimize their care plan [12,34]. Furthermore, AI-driven lifestyle monitoring can provide better oversight for patients living at home, meaning they can potentially remain in their preferred living environments longer [27,41], which is desired by many older adults [42]. Last, positive health revolves around the ability to not focus on the signs, symptoms, and restrictions of disease, but rather to focus on what is possible for the person [43]. AI has the potential to enhance positive health by providing predictive care for older adults [12], thus helping them to maintain or even improve their health.

The potential implications for nurses with the growing integration of AI-driven lifestyle monitoring need attention. Nurses are the largest group of health care professionals worldwide [44], and as such they play a crucial role in the provision of health care. Although it is not commonly expected that AI will replace nurses [10,45], and indeed this is a discussion of complementing nurses rather than replacing them, it is suggested that the dynamics between health care professionals and their patients might be altered by the adoption of AI [46]. For instance, by using AI systems, health care professionals can save time on administrative tasks, thus enhancing efficiency and allowing them to devote more time to establishing trust-based relationships with their patients [20]. Moreover, AI is expected to influence other dimensions of job design such as autonomy, skills, and job demands [20,45]. For example, if AI-driven lifestyle monitoring provides patients with increased information about their health, nurses play a crucial role in guiding and explaining the outcomes to patients, acting as a sort of advisor. As a result, the skills required for health care professionals to effectively interact with both AI systems and increasingly informed patients are undergoing significant changes [20].

## Complementarity Between Nurses and AI-Driven Lifestyle Monitoring

We argue that the qualities of nurses and AI-driven lifestyle monitoring systems in long-term care complement each other, leading to increased value when combined. Nurses excel in the relationship domain, offering emotional support, empathy, and compassion, and working toward the benefit of other humans [13,24], also known as the humane element of nursing and recognized as part of fundamental care [47]. They are good at considering contextual variables to get a holistic view of a patient, are compassionate, and can make genuine connections with the persons for whom they provide care [13]. However, there are limitations to human capabilities. Nurses cannot be present or observe patients around the clock, making it challenging to maintain an objective and comprehensive understanding of a patient’s condition.

On the other hand, AI-driven technologies are particularly skilled at handling tasks that involve analyzing large amounts of data and require substantial computational power [24,36]. AI-driven lifestyle monitoring is capable of identifying long-term behavioral patterns and synthesizing these with data collected from various scenarios. Furthermore, this type of technology can provide continuous monitoring around the clock, even between nursing visits [15,28]. However, AI-driven lifestyle monitoring does lack certain health care–relevant abilities such as dealing with unpredictable situations, considering contextual nuances [36], and the human element of caregiving [16]; skills that are second nature to nurses.

If we were to make use of the qualities of nurses on the one hand and AI-driven lifestyle monitoring systems on the other, we would be able to have the “best of both worlds.” In this situation, we could enrich the nursing caregiving process by adding additional insights from lifestyle monitoring technology and using nursing expertise and patient experience to improve the technology’s practical applications [39] (Figure 1). Next to the human input of the nurse and patient in the care process, a new stream of technical input is provided, formed by the sensors and algorithms of the AI-driven lifestyle monitoring, leading to a visually-presented output [19,28,40] which could provide decision support for nurses [19,40]. For example, sensors could detect disrupted sleep patterns, bathroom use, or changes in how a person moves and walks [27]. If nurses could enrich these findings with nursing expertise and integrate them into clinical knowledge and experience, this would greatly influence the care given. When the output of the sensors is combined with nursing expertise, there is greater potential for care that is better tailored to the current situation and where less time is consumed by gathering information and administration, leaving more time for human contact between the nurse and patient. The AI-driven technical input could be seen as a new digital colleague for the nurse, an idea previously mentioned by Swan [22]. This digital colleague provides deeper insights into the needed care of the older adult and could potentially enhance nurses’ ability to offer more compassionate [13], personalized [12,13,19], and evidence-based [19] care.

## Challenges of the New Digital Colleague

Although we show that cooperation between nurses and AI-driven lifestyle monitoring has promise, it also raises several valid concerns among nurses that should be discussed and considered. First among these is the possibility of decreased patient empowerment and depersonalization, as an overreliance on algorithms could neglect the individual circumstances, preferences, and abilities of the patient [11,12]. In such a situation, actions that are in actuality against the patient’s interest may be justified by the person doing them because they were recommended by the algorithm, as opposed to any normative evaluation by a nurse [11]. At this point, questions of transparency come to mind, as well as the chance of turning care into part of a “black box society,” wherein decisions are made or recommendations are given automatically with limited recourse [17,24]. A lack of algorithmic transparency (ie, poor clarity in how the AI came to the recommendations that it presents) makes it difficult to interrogate those recommendations and decide whether to accept them.

This, in turn, raises a more philosophical, ethical, and methodological concern regarding the use of AI in nursing: given that humanity and human contact have traditionally been seen as a crucial part of the role, there are concerns of this being lost if too much of the care process is based in machines and algorithms [31]. Nurses spend a lot of time interacting directly with patients, often on a personal level. Thus, they are better equipped than most health care professionals to build relationships with those patients and more holistically observe their well-being, meaning they can more easily catch issues that might be missed in clinical assessment [12,31]. As such, it has been argued that overreliance on technology could lead to the dehumanization of patients and overall poorer care [11,12]. Last, on a practical, implementation level, not all nurses currently possess the competence or comfort of working with AI-based systems [24,48,49]. As such, expanded use of AI could lead to more work for nurses, who are often already overextended in their responsibilities [49]. These issues are often exacerbated by poor usability design of the AI interfaces, which may make use of the AI unintuitive and difficult to navigate [10,50].

## How to Collaborate With Our New Digital Colleague

We propose that greater involvement of nurses in the actual design, use, and implementation of AI—in a way, shaping their digital colleague—offers a way to mitigate some of the risks. For example, the nurse’s understanding of patient behaviors and circumstances could act as a sort of counterbalance to the depersonalization of the algorithm [31]. This can be used during the delivery of care, and nurses should certainly be encouraged to not always take the output of the algorithm at face value. However, only having this quality assessment happen at that end point, where nurses have many other tasks and priorities to manage, is not reasonable; far more benefits could be realized by appreciating the role of nurses as knowledge-holders during the algorithm and interface design [1,32,36]. To optimize meaningful health-related features and functionality, it will be necessary to integrate clinical nursing knowledge in the design of the AI. For example, to train AI-driven lifestyle monitoring to identify the early signs of urinary tract infections [27], clinical knowledge provided by nurses should be merged with data from sensors [36]. In short, nurses should be involved in the design process of AI technology [51], also referred to as “nurse-in-the-loop” [27].

Based on their knowledge, nurses may act as advocates for their patients, thereby supporting patient empowerment. Furthermore, with a greater understanding of the AI systems and how the algorithm comes to certain conclusions, nurses are afforded more transparency that they may then pass on to their patients [45]. In practical terms, this would enable them to understand the argumentation of how AI-driven lifestyle monitoring comes to certain conclusions, and thereby could act more critically toward faults or biases. In other words, the much-feared black box society is easier to avoid with a workforce of experienced, knowledgeable nurses who can “shine a light” into that black box [12]. To do this, it is essential to determine the specific competencies required to work with AI-based lifestyle monitoring systems [22,24] and to discuss the responsibilities of individual nurses who work with AI-driven lifestyle monitoring. Nurses should be continuously educated based on these needed competencies [19].

## Conclusion

AI is not, and cannot be, a replacement for nurses. We argue that instead of replacing nurses, AI-driven lifestyle monitoring in long-term care should be seen as a new digital colleague that provides data-based insights to support nursing care. The complementarity of the humane quality of nurses on one side and the AI technology on the other side could lead to more compassionate, personalized, and evidence-based long-term care and can support older adults to age in place. The humane qualities of nurses are enriched by the insights from AI, and vice versa.

This collaboration does come with concerns such as the potential of decreased patient empowerment, depersonalization, and a lack of transparency due to an overreliance on data insights. Furthermore, humanity and human contact could be at stake as the role of AI technology grows. However, these concerns may be addressed with greater nurse involvement. From a practical point of view, to work with AI-driven lifestyle monitoring, specific competencies are required for nurses and the technology should be co-designed in such a way that it fits within nursing workflows. It is therefore crucial to identify the needed competencies to work with AI technology and to gain insight into the needs and wishes of nurses to ensure the design fits within nursing workflows.

## Recommendations

We recommend that long-term care nurses be involved in the actual design, use, and implementation of AI-driven lifestyle monitoring, thus shaping their new digital colleague. This way, nurses can advocate for patient empowerment, add to the transparency of the AI systems, and design the technology to fit within nursing workflows. Furthermore, we recommend prioritizing the development of educational programs to educate our current and future generation nurses to appreciate the potential of AI and be able to collaborate with their new digital colleague.

## Lessons Learned in This Paper

First, AI-driven lifestyle monitoring in long-term care can be seen as a new digital colleague, complementing the qualities of human nurses.

Second, increased use of AI-driven lifestyle monitoring in long-term care comes with some potential risks such as decreased patient empowerment, depersonalization, lack of transparency, and loss of human contact.

Third, the involvement of long-term care nurses in the design, use, and implementation of AI-driven lifestyle monitoring systems could mitigate these challenges.

Finally, AI-driven lifestyle monitoring promises to be a valuable type of AI in long-term care and could potentially enhance long-term care nurses’ ability to offer more compassionate, personalized, and evidence-based care.

## Abbreviations

AI: artificial intelligence
